# Communities of Endophytic Sebacinales Associated with Roots of Herbaceous Plants in Agricultural and Grassland Ecosystems Are Dominated by *Serendipita herbamans* sp. nov

**DOI:** 10.1371/journal.pone.0094676

**Published:** 2014-04-17

**Authors:** Kai Riess, Franz Oberwinkler, Robert Bauer, Sigisfredo Garnica

**Affiliations:** Plant Evolutionary Ecology, Institute of Evolution and Ecology, University of Tübingen, Tübingen, Germany; Graz University of Technology (TU Graz), Austria

## Abstract

Endophytic fungi are known to be commonly associated with herbaceous plants, however, there are few studies focusing on their occurrence and distribution in plant roots from ecosystems with different land uses. To explore the phylogenetic diversity and community structure of Sebacinales endophytes from agricultural and grassland habitats under different land uses, we analysed the roots of herbaceous plants using strain isolation, polymerase chain reaction (PCR), transmission electron microscopy (TEM) and co-cultivation experiments. A new sebacinoid strain named *Serendipita herbamans* belonging to Sebacinales group B was isolated from the roots of *Bistorta vivipara*, which is characterized by colourless monilioid cells (chlamydospores) that become yellow with age. This species was very common and widely distributed in association with a broad spectrum of herbaceous plant families in diverse habitats, independent of land use type. Ultrastructurally, the presence of *S. herbamans* was detected in the cortical cells of *Plantago media, Potentilla anserina* and *Triticum aestivum*. In addition, 13 few frequent molecular operational taxonomic units (MOTUs) or species were found across agricultural and grassland habitats, which did not exhibit a distinctive phylogenetic structure. Laboratory-based assays indicate that *S*. *herbamans* has the ability to colonize fine roots and stimulate plant growth. Although endophytic Sebacinales are widely distributed across agricultural and grassland habitats, TEM and nested PCR analyses reinforce the observation that these microorganisms are present in low quantity in plant roots, with no evidence of host specificity.

## Introduction

Endophytic fungi are phylogenetically diverse and occur nearly ubiquitously in land plants across a variety of ecosystems. At some stage of their complex life cycle, they inhabit plant tissues without causing any obvious disease symptoms [1]. Plant benefits associated with the infection of endophytic fungi include enhanced performance and fitness, stress tolerance and disease resistance [2]–[6]. In recent years, there has been an increasing level of awareness of the diversity and function of fungal endophytes colonizing aboveground plant organs, whereas investigations focusing on the communities inhabiting roots are extremely limited. These few investigations have demonstrated that the diversity and community structure of belowground fungal endophytes are greatly affected by several factors including seasonal changes [7]–[8], the presence of arbuscular mycorrhizal fungi [7], the application of fungicides [9], and the genotype and defence compounds of their hosts [7], [10]. Some but not all fungal endophytes show distinctive host preferences [7] and in contrast to mycorrhizal fungi, many endophytes can be easily cultured which allows subsequent manipulation leading to better a understanding of their ecological functions and interactions.

The first description of a Sebacinales endophyte (*Piriformospora indica*) was carried out by [11] and the details of the mode of root colonization were accurately documented by [12]. Subsequently, more than 150 plant species, including various plants of economic interest like wheat [13], maize [14], tomato [15] and soya bean [16], and several other studies have demostrated the positive effects of endophytic Sebacinales in co-cultivation experiments [17]. Most of these experiments were performed with *P*. *indica* isolated from a desert soil in India and strains of the *Serendipita vermifera* complex isolated from the roots of orchids in Australia, whereas one study has been conducted with the European species *P*. *williamsii* isolated from clover roots [12]. In this study, in order to achieve as complete a picture as possible of the endophytic fungal diversity within the roots of herbaceous plants we used a combination of isolation and polymerase chain reaction (PCR) techniques as suggested by [18].

Although sebacinoid communities are known to be widespread in the field, their composition and function in ecosystems have scarcely been explored. Garnica *et al*. [19] suggested that the diversity, community structure and species composition of endophytic Sebacinales associated with plant communities along an altitudinal gradient can be driven by land use and extremes in soil conditions. Furthermore, the results of this study suggested that Sebacinales are widespread but of low abundance in plant roots, and that some endophytes can colonize multiple host plants. A crucial first step in understanding how these endophyte communities are assembled in plant roots is to characterize their natural composition and occurrence, and subsequently determine whether or not these communities are affected by differences in environmental disturbance or not.

In this paper, our goal was to characterize the patterns of phylogenetic diversity, species distribution and community composition of endophytic Sebacinales from herbaceous plants growing in agricultural and grassland habitats under different land uses. We used a combined approach of strain isolation, microscopy, PCR and co-cultivation experiments to address the following questions: (i) How diverse are endophytic Sebacinales in the roots of herbaceous plants in agricultural and grassland habitats? (ii) To what degree are the diversity and composition of sebacinoid endophyte communities influenced by land use/plant community? (iii) What are the phenotypical effects of a newly isolated sebacinoid strain on selected herbaceous plants? We hypothesized that habitats with similar land use/plant community would show similar endophytic community diversity and composition. In addition, as the newly isolated Sebacinales strain occurs in association with a broad spectrum of herbaceous plants in nature, we used *Arabidopsis thaliana* and *Poa annua* to study the phenotypic response using inoculated and endophyte-free plants.

## Material and Methods

### Ethics statement

Plant species used in this study are not protected and therefore no specific permits were requested for sampling. For sampling in the Natural Reserve “Filsenberg” near Mössingen (Germany), we received permission from the government of Tübingen, Unit Nature Protection and Landscape Conservation (Germany). Individual owners of the agricultural sites gave us permission to collect plant samples for this study.

### Study sites

Herbaceous plants were sampled from various sites surrounding Tübingen, Baden-Württemberg, Germany (Table S1). To more fully determine the phylogenetic diversity and community composition of Sebacinales plant root endophytes, we collected plant material from habitats under different land uses: agricultural habitats (intensive use), adjacent grasslands immediately bordering the agricultural habitats (medium use) and typical grassland habitats (low use). Details concerning land use type and the vegetation composition of the sampled habitats are given in Data S1. Within each habitat, we sampled economically important and co-occurring herbaceous plants (see Table S1). In addition to the individual plants collected in this study, we screened roots from plants where we frequently detected the presence of Sebacinales via PCR [19] as well economically important plants that were not present in the sampled sites. For further diversity and community composition analysis, these were included within the corresponding categories as described above.

### Sampling of plant specimens

In total, we sampled 243 plant specimens from agricultural habitats, 296 from adjacent grasslands and 440 from typical grassland habitats. The entire root system of plant samples and a portion of soil were removed carefully with a shovel and then the roots were soaked in tap water to loosen the soil. Each root sample was rinsed extensively in tap water and then at least three times in distilled water. Using a stereomicroscope, foreign and dead roots were removed with sterile forceps. Subsequently, fine roots were randomly selected and cut with the help of a sterile scalpel for strain isolation and culture, microscopic analysis as well as molecular analysis.

### Isolation and cultivation of sebacinoid endophytes from roots

Root surface sterilization was performed by rinsing the plant material with 30% hydrogen peroxide for 1 min, followed by 70% ethanol for 5 min and finally sterile double-distilled water (2 min). To increase the chance of isolating Sebacinales strains in culture, several media with and without antibiotics/fungicides were used. Root fragments of approximately 0.5 cm length were placed on Petri dishes containing 1.5% water agar, 1.5% Czapek Dox agar, modified Melin Norkrans agar [20], 1.5% malt extract agar or 1.5% potato dextrose agar supplemented with 0.002% streptomycin and 0.005% tetracycline dissolved in 99% ethanol added after autoclaving or with the fungicide Thiabendazole (4 mg/L; Sigma-Aldrich, Steinheim, Germany) [21] or benomyl (2 mg/L; Riedel-de Haen, Seelze, Germany) and Dichloran (2 mg/L; Riedel-de Haen) diluted in 95% ethanol [22]. Root fragments were carefully submerged in the agar with the help of sterile forceps. All the Petri dishes were incubated at 18°C in the dark to promote the growth of endophytes and were periodically checked for any fungus growth. From the non-sporulating fungus colonies that were microscopically characterized by thin, clampless, colourless hyphae, portions of agar and mycelia were transferred to 0.7% malt yeast pepton (MYP) agar. Subsequently, fungal cultures were identified via PCR with specific fungal primers (see below).

### Transmission electron microscopy analyses

To detect morphologically the presence of sebacinoid endophytes in the roots of host plants, we used transmission electron microscopy (TEM). For this evaluation, we screened a total of 150 root samples yielding sebacinoid sequences by TEM examinations following the methodology described in [23].

### DNA extraction, PCR, cloning, sequencing and sequence editing

Total genomic DNA was extracted from fine roots and fungal cultures using the InnuPREP Plant DNA Kit (Analytik Jena, Jena, Germany) or the DNAeasy Plant Mini Kit (Qiagen, Hilden, Germany) following the manufacturer's instructions. Two to three fine roots approximately 1 cm long were selected from each root system, or circa 0.5 cm^2^ of culture medium containing mycelia was placed in Eppendorf tubes. Samples were previously deep-frozen in liquid nitrogen and then ground several times with a sterile plastic pestle.

For the detection of the sebacinoid fungi in root samples, the internal transcribed spacer (ITS1 and ITS2), including 5.8S and the D1/D2 regions of the nuc-rDNA, were amplified using the primer combinations NSSeb1/NLSeb2R [24] with Phusion High-Fidelity DNA Polymerase (Finnzymes Oy, Vantaa, Finland), followed by a nested PCR with ITS1F [25] /NL4 [26] and a Mango*Taq* DNA Polymerase (Bioline, Luckenwalde, Germany) and PCR profiles as described in [19]. For the identification of the fungal cultures, the ITS and the D1/D2 regions were amplified using the primer pair ITS1F and NL4 and Mango*Taq* Polymerase. All PCR products were checked using agarose gel electrophoresis with ethidium bromide staining. PCR products showing multiple bands and those that could not be directly sequenced were cloned using the Topo TA Cloning Kit for Sequencing (Invitrogen, Life Technologies GmbH, Darmstadt, Germany). Fungal colonies were used directly as a template for a PCR with Mango*Taq* Polymerase and the primer pair M13F/M13R (Invitrogen).

The amplified DNA fragments were cleaned using a 1: 20 diluted ExoSAP-IT reagent (USB Corporation, Cleveland, OH, USA). PCR products were cycle sequenced in both directions with the 1: 6 diluted BigDye Terminator v3.1 Cycle Sequencing Kit (Applied Biosystems, Foster City, CA, USA) on an ABI Prism 3130*xl* Genetic Analyzer (Applied Biosystems). Sequence chromatograms were assembled and manually edited using Sequencher 4.10.1 (Gene Codes Corporation, Ann Arbor, MI, USA). All newly generated Sebacinales DNA sequences have been deposited in GenBank and the plants yielding sebacinoid sequences were deposited at the Herbarium Tubingense (TUB) (see Table S1 and Table S3).

### Sequence identity, alignments and phylogenetic analysis

For tentative identification, the ITS sequences of root associated fungi were submitted to BLAST searches against the sebacinoid sequences deposited in the NCBI GenBank database (www.ncbi.nlm.nih.gov). This dataset included a total of 198 ITS+5.8S+D1/D2 LSU sequences, one sequence of the newly isolated Sebacinales strain (*Serendipita herbamans*), 156 newly generated sequences in this study and 41 sequences in total from the studies [19] and [24]. All sequences lacking or having partial ITS1 and/or ITS2 regions and sequences with >3% genetic divergence from our new isolated Sebacinales strain were removed from the final dataset. In addition, to determine the host plant range of *S. herbamans*, we compiled a second dataset with environmental Sebacinales for which large subunit (LSU) sequences are available in GenBank. This dataset included sequences spanning the D1/D2 regions of rDNA with a similarity of 1% [27].

To infer the phylogenetic placement of the newly isolated strain within the order Sebacinales, we assembled two datasets: (i) 35 Sebacinales nuc-rDNA sequences spanning the ITS, 5.8S and D1/D2 regions, and (ii) 48 Sebacinales nuc-rDNA sequences comprising D1/D2 regions from available strains and basidiomata. Because *Serendipita* is not monophyletic and the species delimitations within this genus are still unclear, we have analysed 16 different cultures of the *S. vermifera* complex and also one sequence was selected from each divergent phylogenetic lineage detected in the *Sebacina epigaea* and *S. incrustans* complexes [28].

Sequence datasets were aligned with MAFFT 6.884b using the E-INS-i option [29] and POA 2 under the *-progressive* algorithm [30]. The tool trimAl 1.4 using the algorithm -*compareset* was used to detect the most consistent alignment [31]. Phylogenetic analyses were computed from POA alignments using a maximum likelihood analysis with combined rapid bootstrapping under the GTRCAT model from 1000 runs with RAxML 7.0.4 [32]–[33]. The phylogenetic trees with the best scores were rooted and illustrated using FigTree 1.3 [34].

An uncorrected pairwise distance (*p*-distance) matrix was computed in PAUP* 4.0b10 [35]. Complete ITS sequences with at least 97% similarity were defined as a molecular operational taxonomic unit (MOTU)/species using OPTSIL 1.2 [36].

### Haplotype structure and community composition analyses

To estimate the haplotype phases from the ITS sequences of *Serendipita herbamans*, we used PHASE 2.1 [37]–[38] as implemented in DnaSP 5.10.1 [39] under a Markov Chain Monte Carlo algorithm using 1000 iterations with a hybrid model. The haplotype network was constructed using the median joining method [40] as implemented in Network 4.6.1.2 (www.fluxus-engineering.com) with a ‘MP’ option to identify and eliminate unnecessary median vectors and links [41]. Habitat provenance was coded for each haplotype.

Furthermore, the community structure of Sebacinales endophytes was analysed using Phylocom 4.2 [42]–[43]. To calculate the net relatedness index (NRI) and nearest taxon index (NTI), and to test whether or not endophytic communities have a distinctive phylogenetic structure related to land use, we pooled the samples within the core land use categories as described above. In addition, we compared agricultural habitats + adjacent grasslands vs. typical grasslands, and agricultural habitats vs. adjacent grasslands + typical grasslands. Several null models as implemented in Phylocom were tested. To assign patterns of community structure, we considered NRI and NTI values as significant if values >2 were clustered and <–2 indicated overdispersion, and if both P-values were <0.05 [44].

### Co-cultivation assays and effects on plant phenotypes

In our study, we used *Arabidopsis thaliana* and *Poa annua* because of their small size and because both are easily grown on agar contained in Petri dishes, and because the presence of root-associated Sebacinales endophytes have been detected for both species in the field [24]. To obtain fungus-free *A*. *thaliana* (wild type Columbia Col-0) and *P. annua* (wild type; ProSementis GmbH, Kusterdingen, Germany) seedlings, seeds were washed with double-distilled sterile water, then placed for 1 min in 70% ethanol and 5 min in 1% NaOCl, and finally soaked in double-distilled sterile water for 10 min. The seeds were subsequently placed in ½ Murashige and Skoog (MS) medium [45]. Seed dormancy was broken using a MS medium with the addition of 1% agar, 1% saccharose and 0.01% casamino acids (pH 5.8), followed by incubation period of 48 h at 4°C in the dark. The germination of the seeds was performed at a temperature of 16°C with 8-hour photoperiod.

Prior to the interaction assays, the strains *Piriformospora indica* DSM 11827 and *Serendipita herbamans* DSM 27534 were cultivated on MYP agar for 4 weeks at 18°C in the dark. For each treatment, a total of 40 seedlings of *A. thaliana* and *P. annua* were placed with a sterile tweezers on a MS medium, and an agar block (0.5 cm^2^) containing mycelia of *P. indica* or *S. herbamans* was added near the principal root. Seedlings lacking fungus were used as controls. Interaction experiments were carried out at 16°C with 8-hour photoperiod. The presence of fungal hyphae was analysed using cotton blue in lactic acid under the light microscope. Measurements of the root surface were performed after 24 days for *A. thaliana* and 34 days for *P. annua* using ImageJ 1.45s [46]. Petri dishes containing inoculated and fungus-free seedlings were scanned and aboveground plant parts were cut out from the image. By setting a scale and defining agar, mycelium and Petri dishes as background noise, the root surface for each plant was measured automatically from a planar picture. The fresh and dry weight (dried for 2 days at 60°C) of the roots and shoots were measured and the data were pictured in Origin 9 (ADDITIVE GmbH, Friedrichsdorf, Germany).

### Nomenclature

The electronic version of this article in Portable Document Format (PDF) in a work with an ISSN or ISBN will represent a published work according to the International Code of Nomenclature for algae, fungi, and plants, and hence the new names contained in the electronic publication of a PLoS ONE article are effectively published under that Code from the electronic edition alone, so there is no longer any need to provide printed copies.

In addition, the new species name contained in this work was submitted to MycoBank, from where they will be made available to the Global Names Index. The MycoBank number can be resolved and the associated information viewed through any standard web browser by appending the MycoBank number contained in this publication to the prefix www.mycobank.org/MB. The online version of this work is archived and available from the following digital repositories: PubMed Central, LOCKSS.

## Results and Discussion

### Strain isolation and morphological detection of roots associated Sebacinales

**Figure 1:**
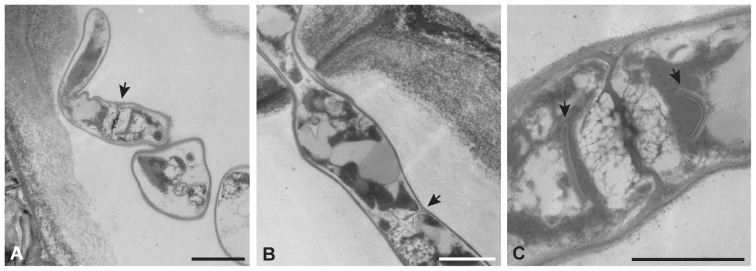
Transmission electron micrographs showing the presence of sebacinoid endophytes in the root cells of *Plantago media.* (**A, B**) Presence of hyphae with a septum (arrows) in cortical cells. (**C**) Septal porus with imperforate parenthesomes (arrows). Scale bar A, B = 1 µm; C = 0.5 µm.

One endophytic Sebacinales strain (MOTU 14, *Serendipita herbamans*) was successfully isolated in water agar medium from non-ectomycorrhizal roots of *Bistorta vivipara* (Polygonaceae). From the 150 root samples yielded sequences of sebacinoid endophytes analysed, was only detected morphologically the presence of Sebacinales in *Plantago media* TUB 019555 (Plantaginaceae, Fig. 1). Previously, the presence of the newly isolated strain was also detected using light and transmission electron microscopy in field samples of *Potentilla anserina* (Rosaceae; [19]) and *Triticum aestivum* (Poaceae; [24]). The hyphae had dolipore septa with imperforate parenthesomes and were present in the outer cortical cells of the fine roots (Fig. 1). Apparently, the cortical cells colonized by the sebacinoid endophytes were not completely vital. In both *P. media* and *P. anserina*, only single hyphae were found; therefore, we cannot make a comparison with the colonization patterns observed in *T. aestivum*. Relevant aspects of the morphological structures, occurrence, phylogenetic relationships and *in vitro* effects on selected herbaceous plants are given below.

### Occurrence, phylogenetic diversity and relationships of Sebacinales root endophytes

**Figure 2:**
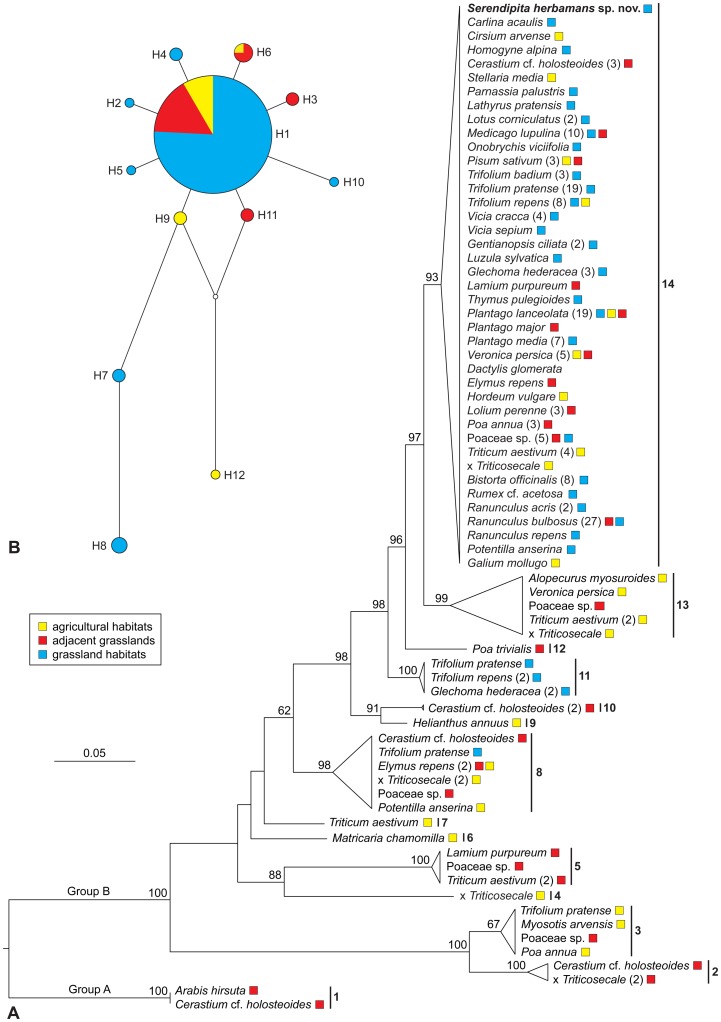
Phylogenetic diversity and relationships of endophytic Sebacinales associated with herbaceous plants in agricultural and grassland habitats. (**A**) Tree topology assessed using a maximum likelihood approach under a GTRCAT model of DNA evolution from 198 sequences spanning the ITS1+5.8S+D1/D2 LSU regions (alignment length = 1300 bp). Numbers above the branches are bootstrap values obtained from 1000 replicates (values ≥60 are shown). Numbers in parentheses indicate the number of sequences found for the same host plant. Bold numbers (1–14) represent MOTUs/species using a 3% cut-off. Coloured boxes indicate land use types (if these were available). Note: MOTU 14 (*Serendipita herbamans*) corresponds to clade 4 in [24] and MOTU 54 in [19]. (**B**) Median-joining network for MOTU 14 (*S*. *herbamans*) inferred from ITS sequences excluding indels [53]–[54] and recombining blocks [55]–[56]. Circle sizes are proportional to haplotype frequency and connecting lines are proportional to mutation events between haplotypes.

Using a nested PCR approach, we were able to generate 156 new rDNA sequences comprising the ITS+5.8S+D1/D2 regions of Sebacinales root endophytes from agricultural and grassland sites in 36 herbaceous species belonging to 11 plant families (Fig. 2A, Table S1). In agreement with [19], [24] and [47], we detected endophytes from plants belonging to Asteraceae, Boraginaceae, Brassicaceae, Caryophyllaceae, Fabaceae, Lamiaceae, Plantaginaceae, Poaceae, Ranunculaceae, Rosaceae and Rubiaceae. Overall, we detected Sebacinales sequences in only 15.9% of the plant roots analysed. The widely sampled families Fabaceae, Plantaginaceae and Ranunculaceae had an above average presence of Sebacinales, whereas Poaceae had a low average presence of the fungi (see Table S2). This is much lower than the 28.9% presence as detected in [47] or 46.5% as reported by [19]. However, these detection rates are difficult to compare because in both studies surveyed, there were more heterogeneous habitats in comparison to agricultural ones. Furthermore, Selosse *et al*. [47] sampled a geographically larger area (Europe and the Caribbean) and Garnica *et al*. [19] also analysed sebacinoids forming mycorrhizae. These results suggest that roots of herbaceous plants in grassland and agricultural ecosystems appear to be sparsely colonized by Sebacinales, which could be partly explained by the influence of intensive land use. Only MOTU 1 clustered in Sebacinales group A, whereas the remaining MOTUs (2–14) are distributed within Sebacinales group B [48]. This is in concordance with previous studies [19], [24] and [47], where most endophytic Sebacinales were included in group B and only a few sequences clustered in group A.

As reported [19], various MOTUs in this study represented singletons: MOTU 4 (x *Triticosecale*), MOTU 6 (*Matricaria chamomilla*), MOTU 7 (*Triticum aestivum*), MOTU 9 (*Helianthus annus*) and MOTU 12 (*Poa trivialis*). The remaining MOTUs were detected in the roots of many different plant species and therefore showed no evidence of host specificity (Fig. 2A). MOTU 14 is described here as a new species, *Serendipita herbamans* (see below), which was the most frequent (76%) endophyte, detected in the roots of 29 herbaceous plant species from eight angiosperm families (Table. S1). We also detected this MOTU in the roots of *Avena sativa* and *Bromus hordeaceus*, but the sequences included only the ITS2 region and therefore, they were removed from the final alignment. Furthermore, based on 1% sequence similarity of the D1/D2 regions of the rDNA, 24 species from 10 plant families represent putative hosts for *S*. *herbamans* (data not shown). This new species has been previously detected via PCR by [19] (MOTU 54) and [24] (clade 4).

In most cases, molecular detection of sebacinoid endophytes requires nested PCRs since morphologically these fungi have been found only in the root cells of three different plant species. This reinforces the observation that this fungal group seems to be relatively widely distributed but of low quantity in plant roots in the field [24], [47], [49].

### Distribution, haplotype and community structure of Sebacinales root endophytes

A total of four MOTUs (4, 6, 7 and 9) were restricted to agricultural habitats and five MOTUs (1, 2, 5, 10 and 12) were found exclusively in adjacent grasslands, whereas one MOTU (11) was detected only in grassland habitats. Considering the low frequency of these MOTUs, we cannot be sure whether or not there is a correlation with land use and/or plant community because the intrinsic nature of these components make it difficult to analyse them separately. On the contrary, MOTUs 8 and 14 (*Serendipita herbamans*) were found to be independent of land use (see Fig. 2A and Table S1). The network analysis for *S. herbamans* resulted in one network with 12 haplotypes: H1 was the most frequent haplotype, and four haplotypes (H2, H5, H10, H12) represented single samples (Fig. 2B). The haplotype H1 was scattered throughout different habitats, whereas the remaining haplotypes were restricted either to adjacent grasslands (H3, H11) or typical grassland habitats (H2, H4, H5, H7, H8, H10). The haplotypes H9 and H12 were restricted to agricultural habitats.

Sebacinoid endophyte communities from agricultural and grassland habitats showed no trend of phylogenetic clustering, whereas adjacent grasslands had a random structure (Tab. 1). Similar endophytic community trends were observed if agricultural habitats and adjacent grasslands or all grassland habitats were pooled together (data not shown). At first glance, this is in contrast to [19], who found significant clustering in root-associated Sebacinales from meadows under different land use intensities. However, if we considered that MOTU 14 (*Serendipita herbamans*) was dominant across all investigated sites (comprising almost 80% of our sequences), it is not surprising that we have not revealed a distinctive phylogenetic structure for the analysed endophytic communities. A similar community structure was observed if the dominant MOTU 14 was excluded from the Phylocom analysis (data not shown). In general, MOTUs/species were more diverse in typical agricultural habitats and adjacent grasslands than in grassland habitats (Table 1). Because our study resulted in a low detection rate (15.9%) of endophytic Sebacinales associated with plant roots, we were not able to compare diversity and community assemblage at a finer scale (e.g. organic vs. no-tillage vs. conventional farming, see Data S1).

**Table 1:** Phylogenetic diversity and structure of endophytic Sebacinales communities associated with herbaceous plants in agricultural and grassland habitats.

Plot	N	S	E	H	D	PD	NRI	NTI	Pattern
Grassland habitats	89	3	0.25	0.28	0.13	0.60	7.95*	−0.66	No trend
Agricultural habitats	29	8	0.79	1.64	0.76	0.28	−2.55	2.31*	No trend
Adjacent grasslands	38	9	0.69	1.51	0.65	0.39	−3.00	0.90	Random

N = Number of sequences, S = MOTUs/species richness, E = Evenness, H = Shannon's diversity index, D = Simpson's diversity index, PD = Faith's index of phylogenetic diversity, NRI = Net Relatedness Index (* = *P*<0.05), NTI = Nearest Taxon Index (*, *P*<0.05). Diversity indexes were calculated as described in [19].

### Phylogenetic placement of *Serendipita herbamans* within Sebacinales

**Figure 3:**
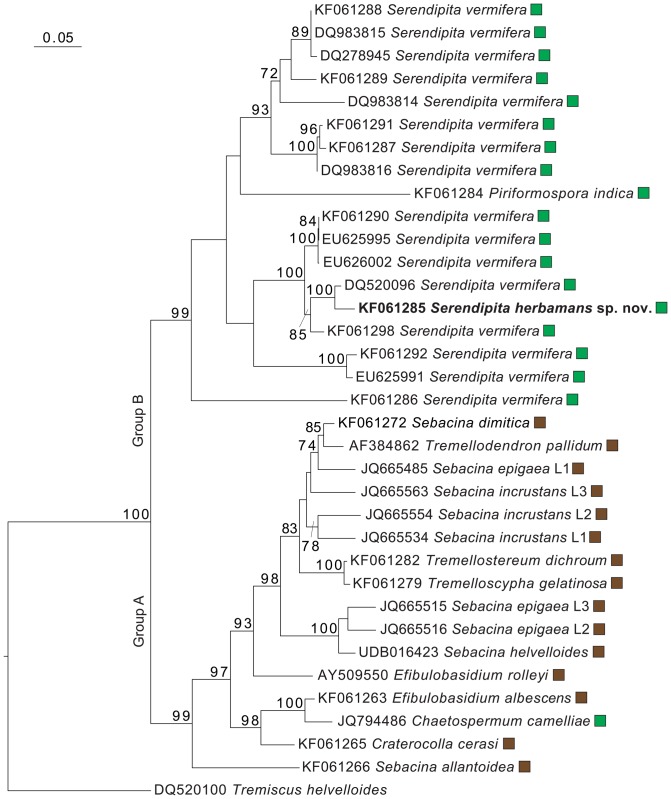
Phylogenetic placement of *Serendipita herbamans* within Sebacinales. Maximum likelihood analysis inferred from 35 ITS+5.8S+D1/D2 rDNA sequences of basidiomata (brown boxes) and culture (green boxes) of the members of the order Sebacinales (alignment length = 1395 bp). *Tremiscus helvelloides* (Auriculariales) was used as an outgroup. Bootstrap supports (≥60%) are shown for each node. For *Serendipita vermifera* MAFF and Warcup's strain numbers [50], see Table S3. L1, L2 and L3 indicate major lineages within *Sebacina epigaea* and *S. incrustans* complexes according to [28].

**Figure 4:**
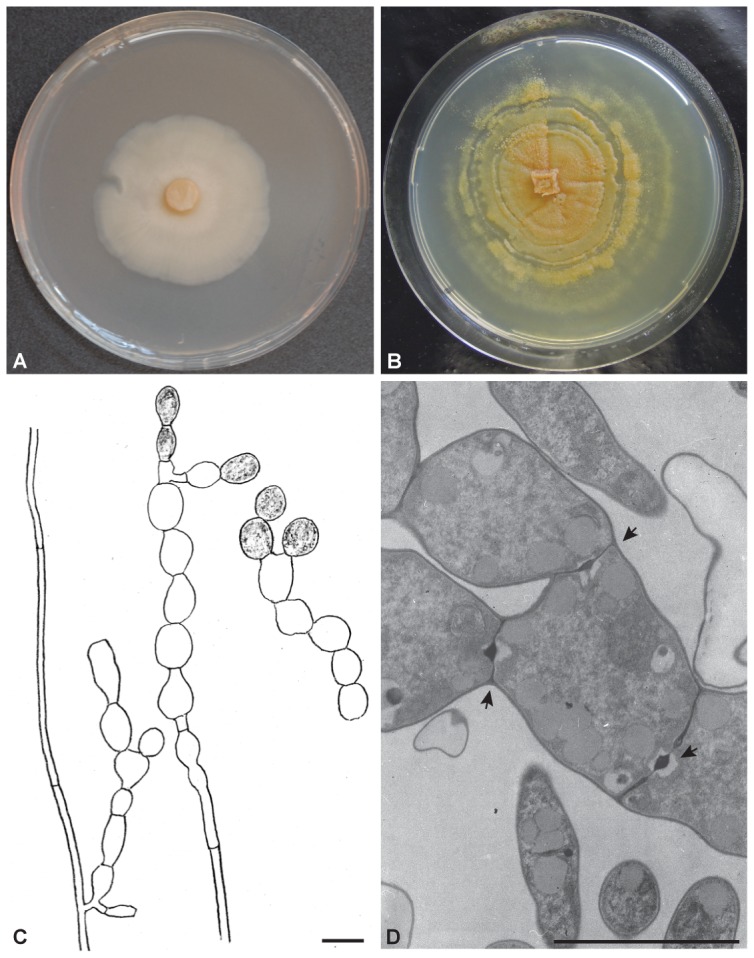
Morphology of *Serendipita herbamans*. (**A**) Colony after two weeks of inoculation on MYP agar. (**B**) Colony after 6 months of inoculation on MYP agar. (**C**) Cylindrical and monilioid hyphae without clamps. Older monilioid hyphae become slightly yellow coloured. (**D**) Transmission electron micrographs showing monilioid hyphae with a septal porus at the constrictions (arrows). Scale bar C, D = 5 µm.

The newly isolated Sebacinales strain (*Serendipita herbamans*) clusters in Sebacinales group B and forms a well-supported clade with some members of the genus *Serendipita* (Fig. 3). The phylogeny based on 34 LSU sequences confirmed the ITS position of *S. herbamans* within the Sebacinales (data not shown). The closest related species to *S*. *herbamans* is *S. vermifera* strain MAFF 305828, which was isolated from the Australian orchid *Eriochilus cucullatus* [50]. *S. herbamans* differs by 29 bp (4.0%) from *S. vermifera* MAFF 305828 in the ITS region. Morphologically, *S*. *herbamans* has a closer similarity with the anamorphic *Piriformospora indica*, but they differ in the size of chlamydospores [11] (see Fig. 4). Phylogenetically, *S*. *herbamans* is distantly separated from *P. indica* with a sequence divergence of 27.7% in the ITS region.

### Interaction assays between Sebacinales strains and herbaceous plants

Co-cultivation of *Serendipita herbamans* with *Arabidopsis thaliana* and *Poa annua* showed that hyphae were abundant on the root surface. After 14 days, the fungus penetrated single root hair cells but only a small portion of the cortical cells in the elongation zone was fully colonized. Hyphae were hardly branched and coiled. The fungus was only detected in the outer cortical cells, but not in the endodermis or the central cylinder. This observation agrees with *in vitro* interaction experiments between *Piriformospora indica* [12], [14], [51] and *S. vermifera* [52] and various host plants. The field colonization pattern of *S. herbamans* in *Triticum aestivum* based on a single snapshot [24] showed a high level of agreement with that in *A. thaliana* and *P. annua* under greenhouse conditions.

**Figure 5:**
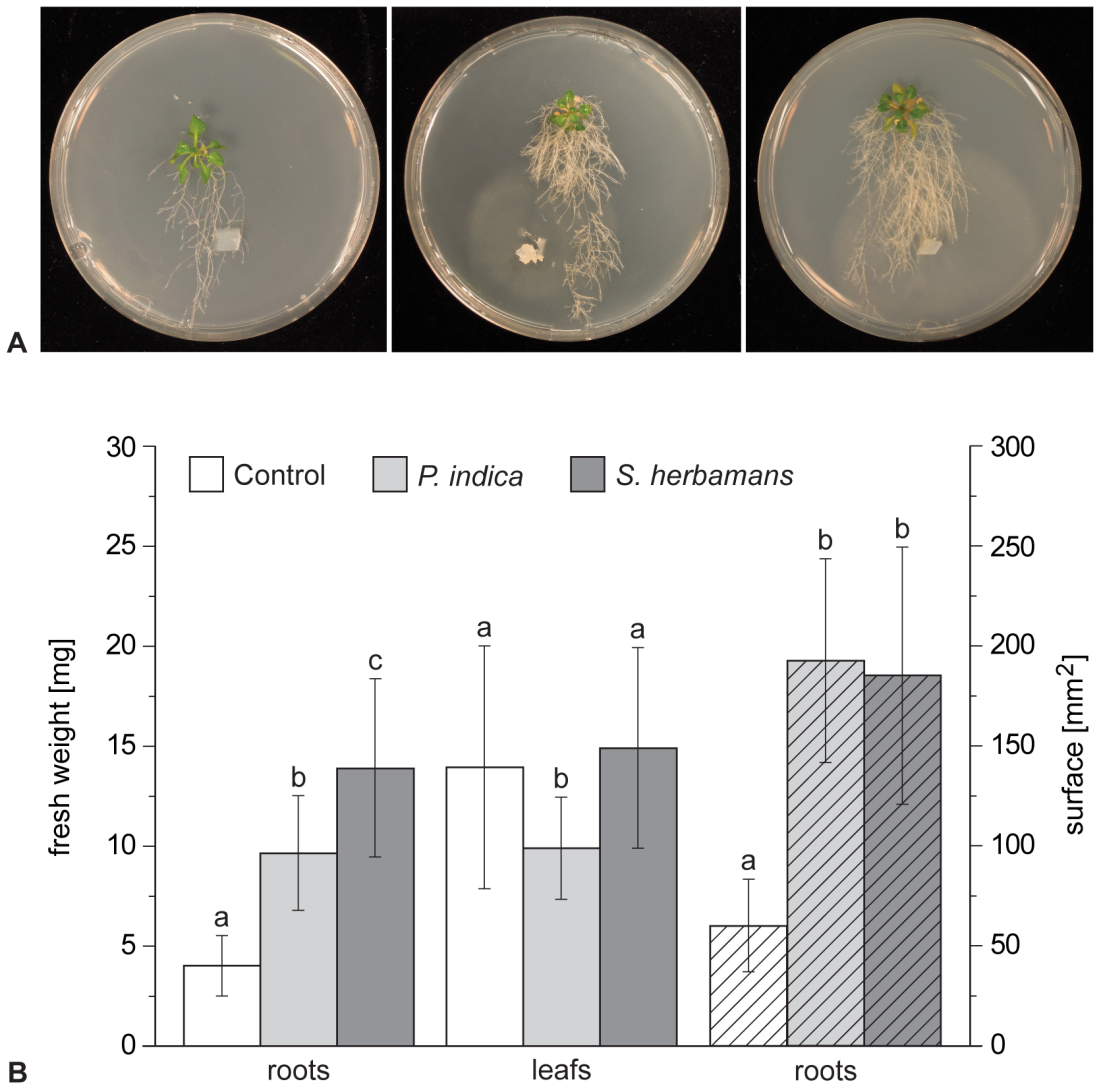
Interaction assays between Sebacinales strains and *Arabidopsis thaliana* under controlled conditions. (**A**) Control (left), inoculated with *Piriformospora indica* (middle) and inoculated with *Serendipita herbamans* (right) seedling phenotypes after 24 days at 16°C. (**B**) Total and root fresh weight and root surface of seedlings after 24 days at 16°C. Error bars give the standard deviation. Different letters indicate significant differences at the confidence level of *P*<0.001 for fresh weight and root surface.

Our experimental interaction assays with *S. herbamans* and *P. indica* demonstrated that these have positive effects on the growth of the roots of *A. thaliana* (see Fig. 5). Seedlings inoculated with the fungi showed stronger root branching compared to the fungal-free plants. The root fresh and dry weight and the root area/surface of *A. thaliana* were significantly higher when grown with Sebacinales than in seedlings lacking of fungi (Fig. 5). On the other hand, no significant differences in leaf fresh and dry weight were observed between inoculated and control seedlings. In addition, *S. herbamans* and *P. indica* were co-cultivated with *P. annua*, confirming the positive effect on the root growth as described for *A. thaliana* (data not shown). Our analyses indicate that *S. herbamans*, like *P. indica* and *S. vermifera* [12], has potential to promote plant growth. Further experiments are planned to address whether *S. herbamans* may also increase plant leaf, seed and/or fruit biomass. The wide occurrence of *S. herbamans,* particularly with economically important plant species from various sites in Europe, opens up huge further potential use of *S. herbamans* as beneficial symbiont in the field.

**Serendipita herbamans K. Riess, Oberw., Garnica, sp. nov.**

(Figs. 1–4)

[MycoBank MB 807011]

### Etymology

Named for its preference to occur in the roots of several herbaceous plant species as revealed via PCR and sequencing.

### Diagnosis

The anamorphic *Serendipita herbamans* forms monilioid hyphae (chlamydospores) (Fig. 4), which have not been reported in the genus until now. Based on the complete ITS region (KF061285), it is phylogenetically closely related to *S. vermifera* strain MAFF 305828 (Fig. 3), but differs from it by 29 bp (4.0%) and *S. vermifera* develops teleomorph stage after a few weeks in culture [50].

### Holotype

Isolated from vital, surface sterilized fine roots of *Bistorta vivipara* (Polygonaceae) growing in a poor meadow lacking ectomycorrhizal trees within a radius of at least 50 m, collected on July 30, 2010 in Bad Hindelang (47°31′ N, 10°24′ E), Bavarian Alps, Germany. The strain has been deposited in Deutsche Sammlung für Mikroorganismen und Zellkulturen (DSMZ), Braunschweig, Germany (DSM 27534).

### Description

Colonies growth rapid on MYP agar, reaching a radius of 13.0±1.2 mm in 14 days at 18°C without light. The fungus has an appressed growth habit and is weakly zonate, and mostly submerged at the edges. It is slightly glossy and cream to ochre-coloured, becoming orange brown with age (Fig. 4A, B). Hyphae were 1–1.5(−2) µm in diameter, cylindrical, thin-walled, clampless and colourless. Young hyphae are cylindrical; with age, these often become septate with constrictions at the septa. This monilioid cells of 3–5 µm×4–5 µm are smooth and colourless, becoming yellow with age (Fig. 4C, D). Monilioid cells (chlamydospores) are more frequent on media with low C and N contents. Basidia and basidiospores were not observed. Hyphae containing typical dolipore septa with imperforate parenthesomes were observed in root cells of *Plantago media* (Fig. 1), *Triticum aestivum* [24] and *Potentilla anserina* [19].

### Geographic distribution

This species has been detected by means of PCR in the roots of 55 species of herbaceous plants belonging to 18 angiosperm families from various agricultural and grassland sites in Europe (Germany, Italy, France) and North America (USA) (Fig. 2A).

## Conclusions

A new endophyte species, *Serendipita herbamans*, is common and widely distributed in plant roots across agricultural and grassland ecosystems. This endophyte grows easily on artificial media and has the potential to promote the growth of herbaceous plants under laboratory conditions. There was no distinctive correlation of diversity and composition of Sebacinales communities with land use in the sampled habitats. Finally, our study indicates that endophytic Sebacinales are widely distributed in the roots of herbaceous plants in agricultural and grassland ecosystems, but they are of low quantity and show no host specificity.

## Supporting Information

Table S1 **Plant species yielding the sebacinoid endophytes detected in this study.** The respective land use category, type of farming/grassland, host plant species, host plant family, sample date, sample location, collector(s), Herbarium Tubingense (TUB) number, GenBank accession number, Sebacinales molecular operational taxonomic unit (MOTU)/species, haplotype designation, sequence source and morphological detection are given. Plant samples TUB 019485–019567 were processed by D. Kuhn in the context of her Diploma thesis.
(XLS)

Table S2 **Total number of herbaceous plant families sampled to detect the presence of sebacinoid fungi in their roots.**
(XLS)

Table S3 **Sebacinales building basidiomata or strains isolated in culture are classified into Sebacinales groups A and B.** The respective GenBank accession number, Herbarium Tubingense (TUB) number/strain designation, collector(s) with collection/isolation number, basidiomata substrate/isolation source and country of collection are given. New sequences in this study use the acronymous KF. Asterisks indicate that only an ITS sequence was available.
(XLS)

Data S1 Description of habitats, including land use and vegetation composition.
(DOC)
