# Supplementary material for: Communities of Endophytic Sebacinales Associated with Roots of Herbaceous Plants in Agricultural and Grassland Ecosystems Are Dominated by Serendipita herbamans sp. nov
Source: PLoS One. 2014 Apr 17;9(4):e94676. doi: 10.1371/journal.pone.0094676 (PMC3990532; doi:10.1371/journal.pone.0094676)
Supplement: Data S1 — Description of habitats, including land use and vegetation composition. (DOC) [file pone.0094676.s004.doc]

The agricultural habitats (intensive use) were divided into three subcategories: (a) organic agriculture, which includes, among other techniques, the use of crop rotation and biological pest control as well as the prohibition of mineral nitrogen fertilizers[[1]](#footnote-2); (b) non-tillage farming, where crop are planted without ploughing, weeds and other competing vegetation are controlled by chemical herbicides, and fertilizers are applied to the soil surface[[2]](#footnote-3); (c) conventional farming, which involves the use of chemical pesticides and chemical fertilizers. Overall, sites with organic agriculture had a greater diversity of co-occurring herbaceous plants as compared to no-tillage and conventional farming sites. The most common co-occurring herbaceous plants in agricultural habitats were those forming rhizomes (*Elymus repens*) or pioneer species (*Alopecurus myosuroides, Convolvulus arvensis,* *Veronica persica*).

In general, adjacent grasslands (medium use) were characterized by soils with high nutrient contents that have been strongly compacted by vehicle traffic. Depending on the type of management, these sites were strongly influenced by the use of fertilizers and pesticides. The adjacent grassland sites had a heterogeneous plant mixture and were mainly dominated by *Agrostis stolonifera*, *Lolium perenne*, *Plantago major,* *Potentilla anserina* and *Trifolium repens,* all of which tolerant of intensive human disturbances.

Grassland habitats (low use) had two subcategories, hay meadows and poor meadows. Hay meadows were characterized by soils with high carbon, nitrogen and phosphate contents. *Arrhenaterum elatius* was the dominant species, accompanied by *Plantago lanceolata*, *Ranunculus acris* and *Trifolium pratense*. Poor meadows were characterized by relatively dry soil with low nutrient contents and high plant diversity, which are partly managed by sheep. These sites were dominated by *Bromus erectus*, while *Potentilla erecta*, *Ranunculus bulbosus* and *Thymus* *pulegioides* also were common species on these sites.

1. Official Journal of the European Union (2007) Council Regulation (EC) No 834/2007 of 28 June 2007 on organic production and labelling of organic products and repealing Regulation (EEC) No 2092/91. Available online at: <http://eur-lex.europa.eu/LexUriServ/LexUriServ.do?uri=OJ:L:2007:189:0001:0023:EN:PDF> [accessed on 24 October 2013]. [↑](#footnote-ref-2)
2. Phillips RE, Blevins RL, Thomas GW, Frye WW, Phillips SH (1980) No-tillage agriculture. Science 208: 1108**–**1113. [↑](#footnote-ref-3)
